# Clinical Profile and Outcomes of Children With Congenital Heart Disease Undergoing Surgery at a Tertiary Care Cardiac Center in Central Gujarat and Their Anthropometric Profiles During Follow-Up: A Prospective Longitudinal Observational Study

**DOI:** 10.7759/cureus.116647

**Published:** 2026-09-21

**Authors:** Naresh D Dhedhi, Vishal V Bhende, Rahul Tandon, Ridham K Nimavat, Amit Kumar, Krutika Tandon

**Affiliations:** 1 Pediatrics, Pramukhswami Medical College, Shree Krishna Hospital, Bhaikaka University, Karamsad, IND; 2 Pediatric Cardiac Surgery, Sri Sathya Sai Sanjeevani Centre for Child Heart Care and Training in Pediatric Cardiac Skills, Kharghar, IND; 3 Pediatric Cardiac Surgery, Bhanubhai and Madhuben Patel Cardiac Centre, Shree Krishna Hospital, Bhaikaka University, Karamsad, IND; 4 Pediatric Intensive Care Unit, Bhanubhai and Madhuben Patel Cardiac Centre, Shree Krishna Hospital, Bhaikaka University, Karamsad, IND

**Keywords:** acyanotic, congenital heart disease, cyanotic, mortality, residual lesions, tetralogy of fallot, ventricular dysfunction, ventricular septal defect

## Abstract

Background

Congenital heart disease (CHD) contributes substantially to pediatric morbidity and mortality. Prospective data describing the clinical profile, in-hospital surgical outcomes, and early postoperative anthropometric trajectory of children treated at cardiac centers outside major metropolitan areas in India remain limited.

Objectives

This study aims to describe the clinical profile and in-hospital outcomes of pediatric patients undergoing surgery for CHD at a semi-urban tertiary cardiac center in central Gujarat and, among survivors, to characterize short-term changes in weight, length/height, body mass index (BMI), and body surface area (BSA) from admission to discharge and at 6-12 weeks after surgery.

Materials and methods

This prospective longitudinal observational study was conducted during 2023 at the Pediatric Division of a semi-urban tertiary cardiac center in Gujarat. Children aged 0-18 years undergoing surgery for CHD were enrolled prospectively and followed from admission through hospitalization and after discharge. Anthropometric measurements were recorded at admission, discharge, and a planned postoperative follow-up between six and 12 weeks. Admission provided the preoperative baseline, discharge represented the immediate postoperative state, and the 6-12-week visit described early post-discharge anthropometric recovery. The short follow-up was not intended to establish sustained catch-up growth.

Results

Among 45 patients, 26 (57.8%) were boys, and 18 (40.0%) were infants. Recurrent respiratory tract infection was the most common presenting symptom (28/45, 62.2%). The three most frequent procedures were ventricular septal defect closure (11 procedures), intracardiac repair for tetralogy of Fallot (10 procedures), and atrial septal defect closure (nine procedures). Six patients died in hospital (13.3%); all six deaths occurred among boys (Fisher's exact test p = 0.032), a finding interpreted as exploratory because of the small number of deaths. Ventricular dysfunction was documented in 34 (75.6%) patients and residual lesions in 22 (48.9%). Among the 39 discharged survivors with complete serial anthropometry, median follow-up was 74 days. Median weight, BMI, and BSA increased slightly by follow-up, whereas length/height changed minimally.

Conclusions

In this single-center cohort, children undergoing CHD surgery had substantial preoperative symptom burden and an in-hospital mortality of 13.3%. Among survivors, weight-related anthropometric measures showed small increases by 6-12 weeks, while length/height changed minimally. These short-term observations describe early postoperative anthropometric trajectory and should not be interpreted as evidence of sustained catch-up growth.

## Introduction

Congenital heart disease (CHD) is an important cause of pediatric morbidity and mortality. Globally, approximately 1.35 million babies are born each year with CHD (about nine per 1000 live births) [1], while Indian studies have reported prevalence estimates of 13.28-26.4 per 1000 patients [2,3]. Access to diagnosis and definitive treatment remains uneven, and pediatric cardiac surgical services are often concentrated in larger urban centers. A recent systematic review of CHD surgery in India highlighted the need for prospective outcome data from diverse Indian centers [4]. Previous studies from Western and Indian settings have reported variable postoperative outcomes after congenital heart surgery. In contrast, differences in patient characteristics, healthcare resources, and perioperative settings may limit direct comparisons across centers [5-7]. Information on postoperative growth and nutritional recovery is also limited, although preoperative nutritional status has been associated with postoperative outcomes and subsequent growth [8,9]. Accordingly, the present study was designed to prospectively describe the clinical profile and in-hospital outcomes of children undergoing CHD surgery at a semi-urban tertiary cardiac center in central Gujarat. A secondary objective was to describe the early postoperative anthropometric trajectory among survivors using repeated measurements of weight, length/height, body mass index (BMI), and body surface area (BSA) at admission, discharge, and 6-12 weeks after surgery. Because this interval is short, the anthropometric component was intended to identify early directional changes rather than establish sustained catch-up growth.

## Materials and methods

This prospective longitudinal observational study was conducted in the pediatric division of a semi-urban cardiac center from January to December 2023. Participants were enrolled before surgery, and clinical, operative, and in-hospital outcome data were recorded forward in time; therefore, the study was prospective. The anthropometric component was longitudinal because the same participants underwent repeated measurements at admission, discharge, and postoperative follow-up. The Institutional Ethics Committee of Bhaikaka University approved the study before enrollment (approval number: IEC/BU/137/Faculty/25/194/2022, dated August 22, 2022). A subsequent amendment concerning the title, short-term follow-up, and addition of a co-author was approved on April 1, 2026 (approval number: IEC/BU/137/Faculty/25/166/2026).

Inclusion and exclusion criteria

As this was a period-dependent study (12 months), no formal sample size calculation was performed. During the study period, the cardiac center had 189 admissions. Of these, 72 were pediatric patients. A total of 45 patients underwent surgery performed by the sole neonatal and pediatric cardiac surgeon from the 63 patients with congenital cardiac defects. Among excluded patients, 12 were stabilized and discharged because an operative procedure was not required, whereas the remaining six were excluded because they had cardiomyopathy and/or congenital heart block.

Data collection

Baseline data included age, sex, residence, clinical findings, diagnosis, type of CHD, anthropometric measurements (weight, length/height, BMI, and BSA), operative procedure, in-hospital outcome, and postoperative complications. Weight was measured using a digital weighing scale, length using an infantometer, and height using a stadiometer. BMI was calculated as weight in kilograms divided by height/length in meters squared, and BSA (m²) was calculated using the Mosteller formula:

$\sqrt{\frac{\text{height/length in cm} \times \text{weight in kg}}{3600}}$

Anthropometric measurements were obtained at admission, at discharge, and at a planned postoperative visit between six and 12 weeks. Admission was selected to document the preoperative anthropometric baseline; discharge documented the immediate postoperative state; and the 6-12-week visit was intended to characterize the direction and magnitude of early post-discharge anthropometric recovery after the acute perioperative period. Given this short interval, we did not expect substantial change in linear growth, and we interpreted length/height descriptively. The schedule was not intended to assess sustained catch-up growth. The primary outcome was in-hospital postoperative status (discharge or death). Secondary outcomes were postoperative complications and the short-term anthropometric trajectory among survivors. The center's sole pediatric cardiothoracic surgeon performed all operations. The center did not have an extracorporeal membrane oxygenation facility. Dedicated cardiac PICU nursing staff and full-time pediatric cardiac intensivists provided postoperative care. Most patients belonged to the lower-middle-income group.

Statistical analysis

Categorical variables were summarized as frequencies and percentages. Anthropometric variables were summarized as median (Q1, Q3) at the three study time points and displayed as boxplots. Patients who died before discharge were excluded from the longitudinal anthropometric analysis; only survivors with complete measurements at all three time points were included, without imputation. Associations of sex with in-hospital outcome and CHD type were assessed using Fisher's exact test because of small cell counts. Analyses were exploratory and unadjusted; p < 0.05 was considered statistically significant. Analyses were performed using Stata (StataCorp LLC, College Station, TX, USA).

## Results

During 2023, 189 admissions occurred at the cardiac center, of which 72 were pediatric patients. Of the 63 children with congenital cardiac defects, 45 underwent cardiac surgery and formed the study cohort. Twenty-six (57.8%) were boys, 18 (40.0%) were infants, and 17 (37.8%) were residents of the local district. Ventricular septal defect was the most frequent diagnosis (15/45, 33.3%), and recurrent respiratory tract infection was the most common presenting symptom (28/45, 62.2%). Postoperatively, ventricular dysfunction was documented in 34 (75.6%) patients, residual lesions in 22 (48.9%), and pulmonary arterial hypertension in 10 (22.2%) (Table 1).

**Table 1:** Baseline characteristics of operated patients (n = 45)

Category	Subcategory	N (%)
Gender	Male	26 (57.8)
Outcome	Discharged	39 (86.7)
	Death	06 (13.3)
Age groups	0-12 months	18 (40.0)
	13-60 months	14 (31.1)
	>5-10 years	09 (20.0)
	>10 years	04 (08.9)
Residence	In one's own district	17 (37.8)
	In one's own state	24 (53.3)
	Outside state	04 (08.9)
Symptoms on presentation	Respiratory tract infection	28 (62.2)
	Poor weight gain/failure to thrive	12 (26.7)
	Cyanotic spells	08 (17.8)
	Breathlessness on exertion	04 (08.9)
	Known case since the neonatal period	03 (06.7)
	Down’s phenotype	01 (02.2)
Diagnosis	Ventricular septal defect	15 (33.3)
	Tetralogy of Fallot	10 (22.2)
	Atrial septal defect	08 (17.8)
	Others	12 (26.7)
Postoperative common issues	Ventricular dysfunction	34 (75.6)
	Residual lesion after surgery	22 (48.9)
	Pulmonary arterial hypertension	10 (22.2)
	Repeat surgery	01 (02.2)

Most CHDs were diagnosed clinically and confirmed by two-dimensional (2D) echocardiography. However, 13 (37.8%) patients also underwent color Doppler (Table 2).

**Table 2:** Operative procedures carried out during the study period (n = 54)* * The same patient may have more than one operative procedure.

Common operative procedures	Frequency	%
Ventricular septal defect closure	11	20.37
Intracardiac repair for tetralogy of Fallot	10	18.52
Atrial septal defect closure	9	16.17
Patent ductus arteriosus ligation and ventricular septal defect closure	4	7.41
Isolated patent ductus arteriosus ligation	3	5.55
Right ventricular outflow tract resection	3	5.55
Redo sternotomy	3	5.55
Aortopulmonary window repair	2	3.70
Completion Fontan	2	3.70
Intraventricular tunnel repair	2	3.70
Pulmonary valvotomy	2	3.70
Complete atrioventricular canal repair	1	1.85
Coarctation of the aorta repair with patent ductus arteriosus ligation	1	1.85
Sinus venosus superior vena cava type atrial septal defect plus partial anomalous pulmonary venous connection re-routing	1	1.85

Table 2 summarizes the operative procedures performed during the study period. Six patients died in hospital (13.3%), and 39 were discharged (86.7%). Among the 39 survivors, 20 (51.3%) were discharged within 10 days, whereas 19 (48.7%) required a hospital stay longer than 10 days. The mean total hospital stay and mean postoperative stay were 15.4 and 12.7 days, respectively. All 39 discharged survivors had complete anthropometric observations at admission, discharge, and follow-up and were included in the longitudinal descriptive analysis. Median follow-up was 74 days (approximately 10.6 weeks), within the planned 6-12-week window. Median (Q1, Q3) weight was 10.0 (6.2, 14.5) kg at admission, 9.5 (6.3, 14.4) kg at discharge, and 10.35 (6.78, 14.75) kg at follow-up. Corresponding median length/height values were 85 (69, 111), 85 (69, 107.5), and 85.45 (70, 105) cm; BMI values were 12.4 (11.3, 13.8), 12.49 (10.72, 13.56), and 13.16 (11.65, 14.05) kg/m²; and BSA values were 0.47 (0.32, 0.64), 0.42 (0.34, 0.67), and 0.49 (0.35, 0.66) m², respectively. At baseline, 11/45 (24.4%) children had weight-for-age within the normal range, and 34/45 (75.6%) had a weight-for-age z-score below -2.

**Figure 1:**
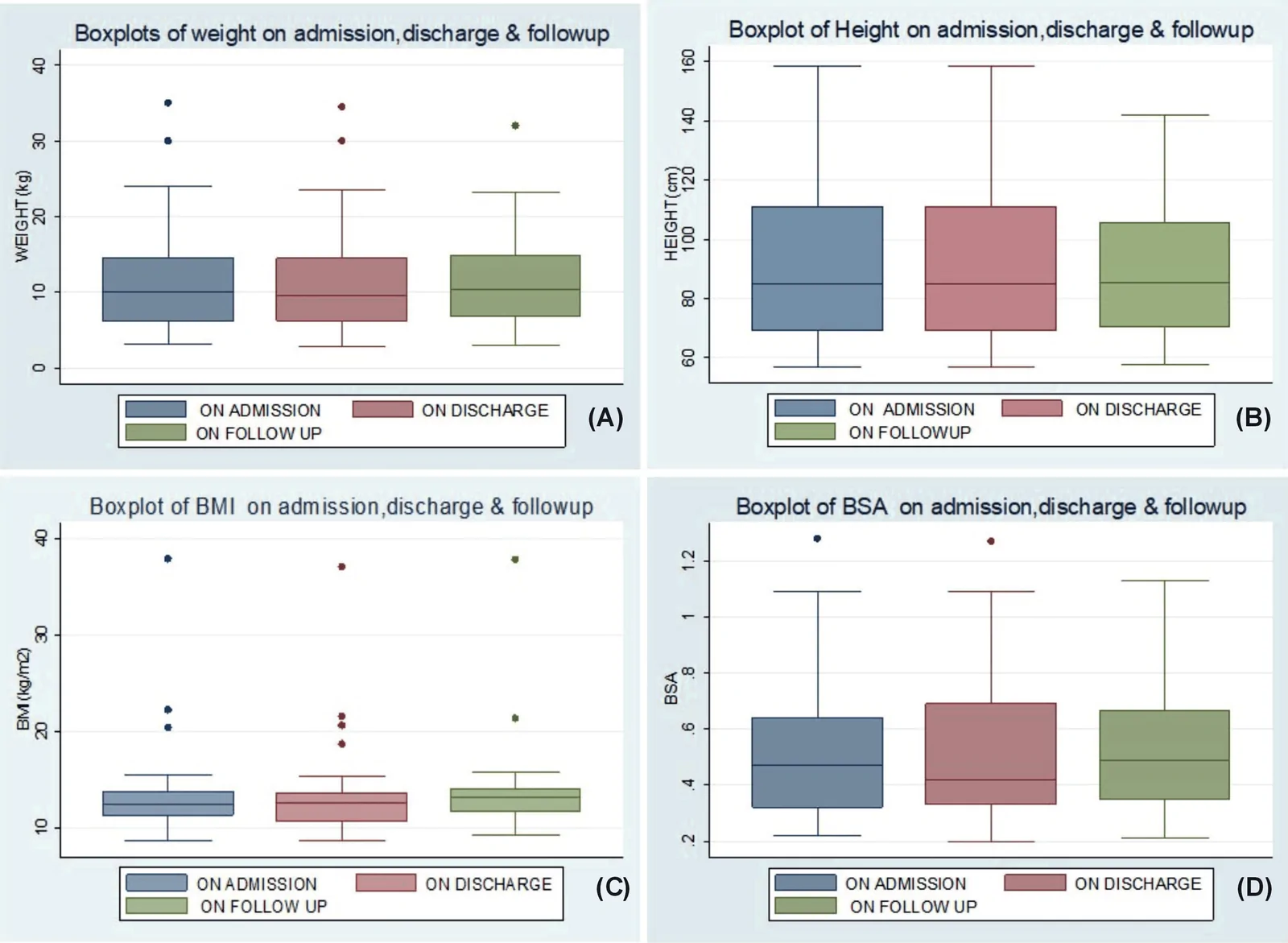
Boxplots of anthropometric measurements in 39 patients with complete observations at admission, discharge, and 6-12-week postoperative follow-up (median follow-up, 74 days) (A) Weight (kg), (B) length/height (cm), (C) BMI (kg/m^2^), (D) body surface area (m^2^) BMI: body mass index, IQR: interquartile range

Figure 1 shows the distributions at the three time points. Median weight decreased slightly by discharge and then increased above baseline by follow-up; BMI and BSA also increased slightly by follow-up, whereas median length/height was essentially stable. Given the short observation period, these findings reflect early postoperative anthropometric recovery rather than evidence of sustained catch-up growth.

The boxplots summarize the distribution of repeated anthropometric measurements in the same 39 survivors. The horizontal line within each box represents the median; the lower and upper box boundaries represent the first (Q1) and third (Q3) quartiles; the box height represents the interquartile range (IQR = Q3-Q1); whiskers extend to the most extreme observations within 1.5 × IQR of the quartiles; and points beyond the whiskers represent outliers. The figure shows the direction, magnitude, and variability of short-term change across the three assessment points, not sustained catch-up growth.

Table 3 presents an exploratory analysis of the association of sex with in-hospital outcome and type of CHD. All six deaths occurred among boys (6/26 boys versus 0/19 girls; Fisher's exact test p = 0.032). Because only six deaths occurred and no adjustment for disease severity, procedural complexity, age, or other potential confounders was possible, this association should be considered hypothesis-generating rather than evidence of a causal sex effect. CHD types did not differ significantly by sex (Fisher's exact test p = 0.194).

**Table 3:** Exploratory association of sex with in-hospital outcome and type of CHD * Fisher's exact test CHD: congenital heart disease

	Gender			p-value*
	Male n = 26	Female n = 19	Total n = 45	
Outcome				
Discharge	20	19	39	0.032
Death	6	0	06	
Type of CHD				
Ventricular septal defect	10	5	15	0.194
Atrial septal defect	3	5	8	
Tetralogy of Fallot	5	5	10	
Complex/other cyanotic CHD	8	2	10	
Ventricular septal defect with patent ductus arteriosus	0	1	1	
Coarctation of aorta with patent ductus arteriosus	0	1	1	

## Discussion

In the present cohort, six of 45 patients died in hospital, corresponding to an observed mortality of 13.3%. This proportion is higher than the pooled mortality reported in the cited systematic review of CHD surgery in India [4]; however, differences in case mix, age distribution, disease complexity, center resources, and the present study's small sample size limit direct comparison. Nine patients underwent one or more concomitant operative procedures, and four of the six deaths occurred in this subgroup (Fisher's exact test p = 0.0103). This finding is exploratory and does not establish that concomitant procedures caused mortality because underlying disease severity and procedural complexity are potential confounders. Ventricular dysfunction was documented in 34 (75.6%) patients and residual lesions in 22 (48.9%), indicating a substantial burden of postoperative morbidity in this cohort. Table 3 also showed that all six deaths occurred among boys (p = 0.032), but the small number of events and absence of multivariable adjustment require cautious interpretation; the finding is hypothesis-generating rather than evidence of a biological or causal sex effect.

The study population was clinically symptomatic at presentation. Recurrent respiratory tract infection was reported in 28 (62.2%) children, poor weight gain/failure to thrive in 12 (26.7%), and cyanotic spells in eight (17.8%). Infants constituted 40.0% of the operated cohort. These findings indicate that many children reached surgery with substantial symptom and nutritional burden. Delayed diagnosis, access to specialized care, socioeconomic barriers, and uneven distribution of pediatric cardiac services have been discussed as challenges in the Indian setting [4,7]; however, the present study was not designed to quantify the contribution of these factors in individual patients.

Postoperative growth and nutritional recovery remain relatively underreported in Indian pediatric cardiac literature. Vaidyanathan et al. described persistent malnutrition during longer follow-up. They emphasized the importance of preoperative nutritional status [8], while other studies have also linked poor preoperative anthropometry with adverse postoperative outcomes [9]. In the present study, anthropometric follow-up was intentionally limited to an early postoperative interval, with a median follow-up of 74 days. As shown in Figure 1, median weight fell slightly between admission and discharge and then increased above the baseline value by follow-up; median BMI and BSA showed similarly small increases, whereas median length/height remained essentially unchanged. The absence of appreciable linear growth over this short interval was expected and reinforces that these measurements should be interpreted as an early postoperative trajectory rather than sustained catch-up growth. The small changes in weight-related measures are descriptive and cannot establish a causal effect of surgery because there was no comparison group, follow-up was brief, and potential confounders such as age, diagnosis, nutritional intake, and disease severity were not adjusted for. A longer follow-up using age- and sex-standardized growth indices is needed to determine whether these early changes translate into sustained nutritional and linear growth recovery.

Patients with even simple CHD who are otherwise healthy have higher morbidity and mortality than the general population [10]. One study of 100 children with unoperated CHD reported malnutrition in 84.0% as compared to 20% of controls. In children with acyanotic CHD, stunting was 57.89%, higher than in cyanotic CHD, whereas wasting was 45.83% in cyanotic CHD. Additionally, malnutrition was more prevalent among children with symptomatic CHD [11]. Using mixed-effects linear models, Aguilar et al. reported that sex and underlying diagnosis affected growth at two years of age. Children with single-ventricle physiology, hypoplastic left heart syndrome, or tetralogy of Fallot frequently exhibit abnormal growth patterns [12]. Factors associated with the need for postoperative rehabilitation include younger age, palliative repair, syndromic conditions, surgery beyond the neonatal period, and a longer preoperative hospital stay [13]. An open chest, single-ventricle physiology, and a shunt may contribute to PICU readmission and prolonged intubation, which can adversely affect postoperative feeding. Thus, feeding difficulties are common in the postoperative period and also affect growth [14]. An Australian study also showed that preadmission growth faltering, the need for tube feeding, cyanotic CHD, and feeding difficulties were associated with hospital stays. Thus, preoperative nutritional screening may help predict postoperative outcomes, and early nutritional intervention may improve patient outcomes [15]. Medoff-Cooper and Ravishankar showed that almost 50% of children younger than one year needed nutritional supplementation via infant feeding or a gastrostomy tube at discharge, especially after univentricular heart defects. Approximately one-quarter of these infants had confirmed failure to thrive. Short stature was also significant among them. They suggested that a structured nutritional program could positively impact growth if implemented at this stage [16]. Among infants with complex CHD, lower weight, length, and head circumference at three months of age and the need for device-assisted feeding have been associated with an increased risk of neurodevelopmental delay in late infancy [17]. Thus, feeding mode and early growth parameters affect growth and developmental outcomes.

The exact mechanism behind this process is also under investigation. In their retrospective matched cohort study, Daymont et al. showed that impairments in different anthropometric growth parameters among children with CHD occurred concurrently rather than sequentially [18]. A multicenter prospective cohort study conducted in China aimed to identify risk factors affecting catch-up growth after surgical intervention in infants with CHD [19]. Cui et al. also called for further research to support early administration of protein- and energy-enriched formula after CHD surgery in infants [20]. A recent study reported that weight was maintained and growth improved among infants managed using a preoperative nutritional pathway. PICU stay was also significantly shorter in the intervention group [21]. Another study highlighted the importance of longitudinal surveillance among children with cyanotic CHD because developmental impairment, growth problems, and reduced quality of life are common concerns in this population [22].

Limitations

Limitations include the single-center design, small sample size, short 6-12-week follow-up, no comparison group, and lack of multivariable analysis. The follow-up permits only a description of early postoperative anthropometric trajectory and cannot assess sustained catch-up growth, particularly linear growth. Discharge weight may be influenced by postoperative illness and perioperative fluid balance. Use of raw anthropometric measures across a wide pediatric age range, without longitudinal age- and sex-standardized z-scores for all measures, also limits growth interpretation. With only six deaths, the observed sex-mortality association is imprecise and cannot be interpreted causally. These factors restrict generalizability.

## Conclusions

In this prospective longitudinal single-center cohort, children undergoing CHD surgery were followed from preoperative admission through in-hospital outcome and a 6-12-week postoperative assessment. In-hospital mortality was 13.3%. Among the 39 survivors with complete follow-up, median weight, BMI, and BSA increased slightly at short-term follow-up, whereas length/height changed minimally. These findings describe early postoperative anthropometric recovery and should not be interpreted as evidence of sustained catch-up growth. The observed association between male sex and mortality is exploratory because of the small number of deaths and lack of adjustment for confounding. Larger multicenter studies with longer follow-up and standardized growth measures are required to define postoperative growth trajectories more reliably.

## References

[1] Hoffman JIe (2013). The global burden of congenital heart disease. Cardiovasc J Afr.

[2] Sawant SP, Amin AS, Bhat M (2013). Prevalence, pattern and outcome of congenital heart disease in Bhabha Atomic Research Centre Hospital, Mumbai. Indian J Pediatr.

[3] Kapoor R, Gupta S (2008). Prevalence of congenital heart disease, Kanpur, India. Indian Pediatr.

[4] Kadiyani L, Kalaivani M, Iyer KS, Ramakrishnan S (2024). The outcome of surgery for congenital heart disease in India: a systematic review and metanalysis. Ann Pediatr Cardiol.

[5] Kempny A, Dimopoulos K, Uebing A (2017). Outcome of cardiac surgery in patients with congenital heart disease in England between 1997 and 2015. PLoS One.

[6] Tandon R, Bhende VV, Kumar A, Dhedhi N, Patel MR, Tandon KR (2025). Clinical profile and outcome of pediatric heart surgeries at semi-urban tertiary care cardiac center of Gujarat, India: a 54-month single-center retrospective chart review. Front Pediatr.

[7] Sharma R (2024). Congenital cardiac surgery outcomes - India versus the West: a case of apples and oranges?. Ann Pediatr Cardiol.

[8] Vaidyanathan B, Nair SB, Sundaram KR, Babu UK, Shivaprakasha K, Rao SG, Kumar RK (2008). Malnutrition in children with congenital heart disease (CHD) determinants and short term impact of corrective intervention. Indian Pediatr.

[9] Lim CY, Lim JK, Moorakonda RB (2019). The impact of pre-operative nutritional status on outcomes following congenital heart surgery. Front Pediatr.

[10] Videbæk J, Laursen HB, Olsen M, Høfsten DE, Johnsen SP (2016). Long-term nationwide follow-up study of simple congenital heart disease diagnosed in otherwise healthy children. Circulation.

[11] Hassan BA, Albanna EA, Morsy SM (2015). Nutritional status in children with un-operated congenital heart disease: an Egyptian center experience. Front Pediatr.

[12] Aguilar DC, Raff GW, Tancredi DJ, Griffin IJ (2015). Childhood growth patterns following congenital heart disease. Cardiol Young.

[13] Ubeda Tikkanen A, Nathan M, Sleeper LA (2018). Predictors of postoperative rehabilitation therapy following congenital heart surgery. J Am Heart Assoc.

[14] Kogon BE, Ramaswamy V, Todd K, Plattner C, Kirshbom PM, Kanter KR, Simsic J (2007). Feeding difficulty in newborns following congenital heart surgery. Congenit Heart Dis.

[15] Costello CL, Gellatly M, Daniel J, Justo RN, Weir K (2015). Growth restriction in infants and young children with congenital heart disease. Congenit Heart Dis.

[16] Medoff-Cooper B, Ravishankar C (2013). Nutrition and growth in congenital heart disease: a challenge in children. Curr Opin Cardiol.

[17] Medoff-Cooper B, Irving SY, Hanlon AL (2016). The association among feeding mode, growth, and developmental outcomes in infants with complex congenital heart disease at 6 and 12 months of age. J Pediatr.

[18] Daymont C, Neal A, Prosnitz A, Cohen MS (2013). Growth in children with congenital heart disease. Pediatrics.

[19] Li L, Li K, An C (2019). Identification of risk factors affecting catch-up growth after infant congenital heart disease surgery: rationale and design of a multicentre prospective cohort study in China. BMJ Open.

[20] Cui Y, Li L, Hu C (2018). Effects and tolerance of protein and energy-enriched formula in infants following congenital heart surgery: a randomized controlled trial. JPEN J Parenter Enteral Nutr.

[21] Marino LV, Johnson MJ, Davies NJ (2020). Improving growth of infants with congenital heart disease using a consensus-based nutritional pathway. Clin Nutr.

[22] Maya S, Gunawijaya E, Yantie NP, Windiani IG (2020). Growth, development, and quality of life in children with congenital heart disease children. Open Access Maced J Med Sci [Internet].

